# Diabetic Foot Syndrome and Corneal Subbasal Nerve Plexus Changes in Congolese Patients with Type 2 Diabetes

**DOI:** 10.1371/journal.pone.0119842

**Published:** 2015-03-26

**Authors:** Andrey Zhivov, Sabine Peschel, Hans-Christof Schober, Oliver Stachs, Simone Baltrusch, Marie Therese Bambi, Janvier Kilangalanga, Karsten Winter, Guenther Kundt, Rudolf F. Guthoff

**Affiliations:** 1 Department of Ophthalmology, University of Rostock, Rostock, Germany; 2 Clinic of Internal Medicine I, Klinikum Südstadt, Rostock, Germany; 3 Institute of Medical Biochemistry and Molecular Biology, University of Rostock, Rostock, Germany; 4 Department of Ophthalmology, St. Joseph Hospital, Kinshasa, Democratic Republic of Congo; 5 Translational Centre for Regenerative Medicine (TRM), University of Leipzig, Leipzig, Germany; 6 Institute for Biostatistics and Informatics in Medicine and Ageing Research, University of Rostock, Rostock, Germany; UCLA School of Medicine, UNITED STATES

## Abstract

**Background:**

To study the severity of diabetic neuropathy, diabetic retinopathy and grades of diabetic foot syndrome for correlations with corneal subbasal nerve plexus (SBP) changes in Congolese patients with type 2 diabetes.

**Methodology/Principal Findings:**

Twenty-eight type 2 diabetes patients with diabetes-related foot ulceration were recruited in a diabetic care unit in Kinshasa, Democratic Republic of Congo. Corneal SBP was investigated by confocal laser-scanning microscopy to analyse nerve fibre density (NFD) [µm/ µm²], number of branches [n] and number of connectivity points [n]. Foot ulceration was graded using the Wagner ulcer classification. Corneal sensitivity (Cochet-Bonnet), Neuropathy Symptom Score (NSS), Neuropathy Disability Score (NDS), ankle-brachial index (ABI) and ophthalmological status were evaluated. Foot ulceration was ranked as mild (Wagner 0-1: 13 patients/46.4%), moderate (Wagner 2-3: 10 patients/35.7%) and severe (Wagner 4-5: 5 patients/17.9%). The correlation between Wagner Score and NFD (p=0.017, r = - 0,454), NDS and NFD (p=0,039, r = - 0.400) as well as Wagner Score and HbA_1c_ (p=0,007, r = - 0.477) was stated. Significant differences in confocal SBP parameters were observed between Wagner 0-1 and Wagner 4 5 (number of branches (p=0.012), number of connectivity points (p=0.001), nerve fibre density (p=0.033)) and ABI (p=0.030), and between Wagner 2-3 and Wagner 4-5 (number of branches (p=0.003), number of connectivity points (p=0.005) and nerve fibre density (p=0.014)). Differences in NDS (p=0.001) and corneal sensation (p=0.032) were significant between Wagner 0-1 and Wagner 2-3. Patients with diabetic retinopathy had significantly longer diabetes duration (p=0.03) and higher NDS (p=0.01), but showed no differences in SBP morphology or corneal sensation.

**Conclusions/Significance:**

While confirming the diabetic aetiology of foot ulceration due to medial arterial calcification, this study indicates that the grade of diabetic foot syndrome correlates with corneal SBP changes and corneal sensation in patients in sub-Saharan Africa.

## Introduction

The International Diabetes Federation has estimated that 4.3% of adults in the Africa Region suffer from diabetes mellitus (DM). The Democratic Republic of Congo (DRC) ranks fourth in the top ten countries by diabetes cases in Africa [1]. Lack of financial support makes effective diagnosis and therapy impossible: some 48.68% of type 2 DM patients in DRC are uncontrolled, and the resulting diabetic neuropathy (DN) is the most common long-term complication of DM [2]. DN is also a leading cause of the foot ulceration encountered in DM patients. About 15% of DM patients develop foot ulceration, and about 50% of diabetes-related in-patient treatment is accounted for by diabetic foot ulceration. Recent research has described the development of foot ulceration as a consequence of DN and peripheral vascular disease [3].

Peripheral DN affects the Aδ and C types of small nerve fibres. The current gold standard for evaluating small-fiber neuropathy is intradermal nerve fibre density measurement in skin biopsy specimens. In-vivo confocal microscopy permits assessment of the unmyelinated fibres of the corneal subbasal nerve plexus (SBP) and has emerged as a non-invasive and reliable option for evaluating small-fibre neuropathy. A complex relationship has been demonstrated between SBP impairment, decreased corneal sensation and the development of diabetic retinopathy (DR) and DN [4].

Within the framework of treatment and surveillance of DM patients in the Department of Ophthalmology at Saint Joseph Hospital in Kinshasa a major discrepancy was noted between the presence of extensive foot ulcerations yet only mild to moderate diabetic retinopathy in most patients [5]. To our knowledge only very few publications have addressed the issue of diabetic foot syndrome in Africa. Using the established diagnostic modalities for the first time in a sub-Saharan Africa setting, the logical next step was therefore to elucidate the aetiology of this condition and to establish possible correlations between the severity of peripheral DN, corneal neuropathy and DR.

## Materials and Methods

### Patients

This study was conducted after a positive appraisal by the ethics review board of Rostock University (permit number: A 2012–0118). The research adhered to the tenets of the Declaration of Helsinki. The authorities of St. Joseph Hospital in Kinshasa (DRC) also granted permission for this study in writing, and it was conducted in February 2012 in that institution by a combined team of internists and ophthalmologists. Patients with type 2 DM scheduled to attend the local outpatient department were enrolled in the study. A detailed consent review was carried out and the participants’ informed consent was obtained in writing. Data were collected by interview, review of patients’ medical records and investigation. We certify that all applicable institutional and governmental regulations concerning the ethical use of human volunteers were followed during this research.

### General clinical status

The patients’ demographic data were collected, together with details concerning the onset date of diabetes or symptoms, recorded diabetes type, current diabetes management and treatment.

Glycaemic control was assessed with reference to HbA_1c_ and blood sugar (DCA 2000+ capillary blood glucometer; Siemens, Eschborn/Germany). The diagnosis of DM was based on the American Diabetes Association (ADA) recommendation of a random or casual blood sugar level ≥ 200 mg/dl (11.1 mmol/l) [6]. Type 2 DM was verified in patients aged over 30 years who were receiving oral hypoglycaemic drugs and/or insulin therapy.

The Neuropathy Symptom Score (NSS) was performed as described elsewhere [7] and classified as ‘no neuropathy’ (0–2 points), ‘mild neuropathy’ (3–4 points), ‘moderate neuropathy’ (5–6 points), and ‘severe neuropathy’ (>6 points) [8]. The Neuropathy Disability Score (NDS) included testing with the tuning fork, 10-g monofilament (large-fibre function), and temperature perception with hot/cold rods (small-fibre function), as described by Quattrini *et al*. [9]. A score of 3–5 was regarded as evidence of mild, 6–8 as moderate, and 9–10 as severe signs of neuropathy [8].

The ankle-brachial index (ABI) was evaluated to screen the patients for peripheral vascular disease (PVD). The ABI was obtained by measuring systolic blood pressures in the ankles (dorsalis pedis and posterior tibial arteries) and arms (brachial artery) using a handheld Doppler (Huntleigh Dopplex D900, Cardiff/United Kingdom) and then calculating the ratio [10]. An ABI <0.9 was interpreted as denoting the presence of PVD.

Foot ulceration was graded using the Wagner ulcer classification based on wound depth and the extent of tissue necrosis [11]. Ulceration was recorded with a tape measure and photographed with a digital camera. The Armstrong classification [12] based on the infection component in ulcer development was not used due to very poor local hygiene standards (barefoot patients or low-grade foot wear) and the lack of facilities for microbiological/histological specimen analysis.

Although possible in theory, electrophysiological investigations to identify surrogate endpoints for foot ulceration and evaluations of small-fibre neuropathy using skin punch biopsy have not yet been performed in sub-Saharan Africa to our knowledge. Our investigations also included in-vivo CLSM of the SBP as a clinically established parameter which correlates well with PDN and has the potential to replace time- and resource-consuming electrophysiology or skin biopsy testing

### Ophthalmological status

All patients underwent slit-lamp investigation including funduscopy in mydriasis. The staging of diabetic retinopathy was performed as described elsewhere [13]. All investigations were performed by a single ophthalmologist masked to the clinical history (SP).

#### Corneal sensation

Corneal sensation was measured with a Cochet-Bonnet aesthesiometer in the central and peripheral cornea (Luneau Ophthalmologie, Paris/France; monofilament diameter 0.12 mm). The monofilament was applied along its full length of 60 mm; if the response was negative, the length was subsequently reduced in 5 mm steps until a positive response was achieved.

#### *In-vivo* confocal laser-scanning microscopy (CLSM)

The patients’ corneas were investigated in vivo using the Heidelberg Retina Tomograph (HRT II) in combination with the Rostock Cornea Module (RCM) (Heidelberg Engineering, Heidelberg/Germany). The HRTII/RCM system uses a diode laser source with a wavelength of 670 nm and is equipped with a water contact objective (63x/0.95W, 670 nm; Zeiss, Jena/ Germany). The distance from the cornea to the microscope was kept stable by a single-use contact element in sterile packaging (Tomo-Cap; Heidelberg Engineering, Heidelberg/Germany). Coupling between the patient’s cornea and the cap was facilitated with a thin lubricant layer of Vidisic gel (Bausch & Lomb/Dr. Mann Pharma, Berlin/Germany; refractive index 1.35). The eye to be examined was anaesthetised by instilling Proparakain 0.5% eye drops (Ursapharm, Saarbrücken/Germany).

Image acquisition of the central cornea was performed in z-scan of automatic volume scan mode (30 images, volume depth 60 μm, constant interslice distance 2 μm). The acquired images have a definition of 384 x 384 pixels over an area of 400x400 μm.

Confocal microscopy was performed in the region of interest, i.e. at the level of basal cells, SBP, Bowman’s membrane and anterior stroma at depths from 30 to 90 μm. At least three scans were performed. The total duration of in-vivo CLSM was about 15 minutes.

#### Quantification of micromorphological parameters

The evaluation of SBP on the basis of a best artefact-free single image was performed automatically on pre-segmented images using a software tool developed in house [14] and was based on morphological (length, diameter, density) and topological (continuity and connectivity) parameters. In order to illustrate the most representative data the number of analysed parameters was reduced to those displaying significant between-group differences (p<0.05). The current study therefore focused on evaluating average single fibre length [μm], nerve fibre density [μm/μm^2^], number of connectivity points [n] and number of branches [n].

### Statistical analysis

For processing and statistical analysis of all data, the SPSS software package, version 21.0 (SPSS GmbH, Munich, Germany), was used.

Descriptive statistics were computed for continuous and categorical variables. The statistics computed included mean and standard deviations (SD) of continuous variables, frequencies and relative frequencies of categorical factors and are presented as mean ± SD.

Because of the small number of subjects and the low power of a test on normality comparisons between groups were done by using nonparametric Kruskal-Wallis test or Mann-Whitney *U* test by ranks and correlations were computed by using Pearson’s correlation coefficient.

All p-values resulted from two-sided statistical tests and values of p < 0.05 were considered to be statistically significant.

## Results

Twenty-eight patients with type 2 DM and diabetes-related foot ulceration (12 male/16 female, aged 59 ± 8.1 years) were recruited from the patients pool of St. Joseph Hospital in Kinshasa/DRC. The mean duration of DM was 13.4 ± 7.4 years. Mean ABI was 1.1 ± 0.3, thus emphasizing the medial arterial calcification as the primary reason in ulcer causation. Peripheral DN was characterised by an NSS score of 5.9 ± 2.8 points and an NDS score of 6.5 ± 3.0 points indicating moderate to severe peripheral neuropathy. The patients’ demographic and clinical data are presented in Table 1.

**Table 1:** Clinical and demographic data for all participants and for various subgroups (data presented as mean ± SD).

Group	All (n = 28)	NDR (n = 11)	DR (n = 17)	Wagner 0 (n = 9)	Wagner 1–5 (n = 19)	Wagner 1–5+DR (n = 13)	Wagner 1–5 +NDR (n = 6)	Wagner 0+NDR (n = 5)	Wagner0–1 (n = 13)	Wagner2–3 (n = 10)	Wagner4–5 (n = 5)
**Gender [m/f]**	12/16	5/6	7/10	6/3	6/13	4/9	2/4	3/2	8/5	3/7	1/4
**Age [years]**	59±8.1	60.1±7.3	58.2±8.7	57.9±6.2	59.5±9	58.2±9	62.3±9.2	57.4±3.4	57.2±6.2	63.4±6.7	54.6±12
**Duration of DM [years]**	13.4±7.4	10.5±7.6	15.3±7	15±11.3	12.7±4.9	14.3±5	9.2±2.1	12.2±11.4	13.9±9	14.1±7	10.8±2.8
**HbA**_**1c**_ **[%]**	10.7±2.5	9.8±2.1	11.2±2.6	10.2±3.2	10.9±2.0	11.4±2.1	9.8±1.5	9.8±2.8	11.2±2.4	10.4±2.7	9.8±2.1
**NSS [points]**	5.9±2.8	6.6±1.6	5.5±3.3	5.1±3.6	6.3±2.3	6.1±2.6	6.8±1.3	6.4±2.1	6.2±2.9	5.4±3.3	6.4±0.9
**NDS [points]**	6.5±3.0	5.3±2.8	7.3±2.9	5.1±3.1	7.1±2.8	7.3±3.1	6.8±1.7	3.4±2.8	5.0±2.6	8.6±1.8	7.4±1.9
**Corneal sensation [mm]**	44±20	49±22	42±19	54±11	40±22	40±20	40±28	60±0	55±10	36±24	4.6±2.1
**ABI(ulcer)**	1.1±0.3	1.±0.4	1.1±0.2	1.1±0.2	1.1±0.3	1.2±0.2	0.9±0.5	1.2±0.2	1.0±0.3	1.1±0.3	1.3±0.1
**Nerve fibre density [μm/μm^2^]**	0.015±0.008	0.016±.007	0.014±0.008	0.017±0.007	0.014±0.009	0.014±0.009	0.013±0.007	0.02±0.005	0.018±0.01	0.014±0.004	0.008±0.003
**Nerve branches [n]**	180.7±152.3	185.9±03.4	177.3±180.1	198.9±99.1	172±173.7	187.3±20.2	138.8±98	242.4±86.1	237.4±197.4	163.1±74.6	68.3±11.8
**Connectivity points [n]**	19.8±12.9	18.6±9.2	20.6±15.1	23.7±11.7	17.9±13.4	19±15.5	15.6±7.7	22.1±10.4	24.7±14.3	19.6±10.4	7.4±2.6
**Average single fibre length [μm]**	22.4±5.5	25.2±4.5	20.5±5.3	22.6±4.1	22.3±6.1	20.8±6	25.5±5.6	24.9±3.5	23.6±5.1	20.7±5	22.7±7.4

ABI = Ankle-Brachial Index; DR = Diabetic retinopathy; NDR = No diabetic retinopathy; NDS = Neuropathy Disability Score; NSS = Neuropathy Symptom Score.

The DM patients were divided into two subgroups according to the absence (NDR, 11 patients, 39.3%) or presence (DR, 17 patients, 60.7%) of diabetic retinopathy [13]. These two subgroups differed in duration of diabetes (p = 0.027) and NDS (p = 0.011), but not in HbA_1c_ and age. Corneal sensation in the two subgroups did not differ significantly.

**Fig 1:**
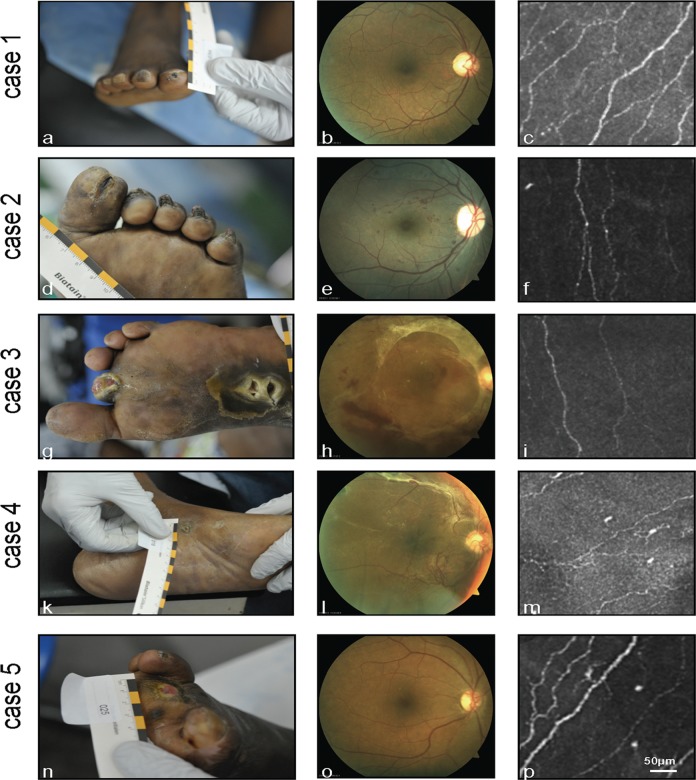
Representative cases. Case 1: 62-year-old male patient, DM duration 9 years, HbA_1c_ 6.5%, NDS 4 points; Wagner 1 (a), no signs of DR (b), normal SBP morphology (NFD = 0.011 μm/μm^2^) (c). Case 2: 62-year-old female patient, DM duration 7 years, HbA_1c_ 9.4%, NDS 5 points; Wagner 2 (d), advanced non-proliferative DR (e), moderate decrease in confocal SBP parameters (NFD = 0.010 μm/μm^2^) (f). Case 3: 44-year-old female patient, DM duration 9 years, HbA_1c_ 11%, NDS 9 points; Wagner 5 (g), proliferative DR with retinal detachment (h), moderate decrease in confocal SBP parameters (NFD = 0.011 μm/μm^2^) (i). Case 4: 59-year-old female patient, DM duration 31 years, HbA_1c_ 10.4%, NDS 6 points; Wagner 0 (k), proliferative DR (l), moderate decrease in confocal SBP parameters (NFD = 0.008 μm/μm^2^) (m). Case 5: 58-year-old female patient, DM duration 12 years, HbA_1c_ 8.2%, NDS 5 points; Wagner 5 (n), no signs of DR (o), normal SBP morphology (NFD = 0.014 μm/μm^2^) (p)

The same patients were categorised into three subgroups depending on severity of foot ulceration (Fig. 1): mild (Wagner 0–1: 13 patients, 46.4%), moderate (Wagner 2–3: 10 patients, 35.7%) and severe (Wagner 4–5: 5 patients, 17.9%). These subgroups did not differ in terms of DM duration, HbA_1c_ and age. The NDS Score was significantly increased (p = 0.001) and corneal sensation significantly decreased (p = 0.032) in the comparison between Wagner 2–3 and Wagner 0–1. ABI was significantly higher (p = 0.03) in the comparison between Wagner 4–5 and Wagner 0–1.

The comparison between patients with Wagner 0 (pre-ulcerative site, or healed ulcer) and Wagner 1–5 (any ulcer) revealed only an increase in NDS. The comparison of Wagner 1–5 patients with DR with Wagner 0 patients without DR showed increased NDS and decreased corneal sensation.

In-vivo CLSM revealed alterations in SBP morphology between the different groups (Fig. 1). Qualitatively, there seemed to be a reduction in nerve fibre and branch density in Wagner 2–3 and 4–5 compared to Wagner 0–1. To verify these qualitative findings, further detailed image analysis was conducted (cf. Methods) and the results are presented in Tables 1–3.

**Table 2:** Correlation between study parameters in diabetic patients.

		Duration of DM [years]	HbA1c [%]	Nerve fibre density [μm/μm2]	Wagner-Score	NDS [points]	NSS [points]
**Duration of DM [years]**	Correlation—Pearson	1	,061	,127	-,244	,208	-,308
	Significance (bilateral)		,743	,526	,185	,261	,091
**HbA1c [%]**	Correlation—Pearson	,061	1	-,186	-,477**	,227	,112
	Significance (bilateral)	,743		,352	,007	,219	,548
**Nerve fibre density [μm/μm^2^]**	Correlation—Pearson	,127	-,186	1	-,454*	-,400*	-,171
	Significance (bilateral)	,526	,352		,017	,039	,394
**Wagner-Score**	Correlation—Pearson	-,244	-,477**	-,454*	1	,115	-,009
	Significance (bilateral)	,185	,007	,017		,538	,960
**NDS [points]**	Correlation—Pearson	,208	,227	-,400*	,115	1	-,016
	Significance (bilateral)	,261	,219	,039	,538		,930
**NSS [points]**	Correlation—Pearson	-,308	,112	-,171	-,009	-,016	1
	Significance (bilateral)	,091	,548	,394	,960	,930	

** Correlation significant 0,01 (bilateral);

* correlation significant 0,05 (bilateral)

**Table 3:** Differences between study parameters.

**Differences**	**Duration of DM**	**HbA**_1c_	**NSS**	**NDS**	**Corneal sensation**	**ABI (ulcer)**	**Nerve fibre density**	**Nerve branches**	**Connectivity points**	**Average single fibre length**
**NDR** ↔ **DR**	0.027	n.s.	n.s.	0.011	n.s.	n.s.	n.s.	n.s.	n.s.	0.023
**Wagner 0** ↔ **Wagner 1–5**	n.s.	n.s.	n.s.	0.026	n.s.	n.s.	n.s.	n.s.	n.s.	n.s.
**Wagner 1–5 +DR** ↔ **Wagner 1–5 +NDR**	0.029	n.s.	n.s.	n.s.	n.s.	n.s.	n.s.	n.s.	n.s.	n.s.
**Wagner 1–5 +DR** ↔ **Wagner 0 +NDR**	n.s.	n.s.	n.s.	0.007	0.016	n.s.	0.043	n.s.	n.s.	n.s.
**Wagner 0–1** ↔ **Wagner 2–3**	n.s.	n.s.	n.s.	0.001	0.032	n.s.	n.s.	n.s.	n.s.	n.s.
**Wagner 0–1** ↔ **Wagner 4–5**	n.s.	n.s.	n.s.	n.s.	n.s.	0.030	0.033	0.012	0.001	n.s.
**Wagner 2–3** ↔ **Wagner 4–5**	n.s.	n.s.	n.s.	n.s.	n.s.	n.s.	0.014	0.003	0.005	n.s.

ABI = Ankle-Brachial Index; DR = Diabetic retinopathy; NDR = No diabetic retinopathy; NDS = Neuropathy Disability Score; NSS = Neuropathy Symptom Score; n.s. = non-significant (p>0.05)

**Fig 2:**
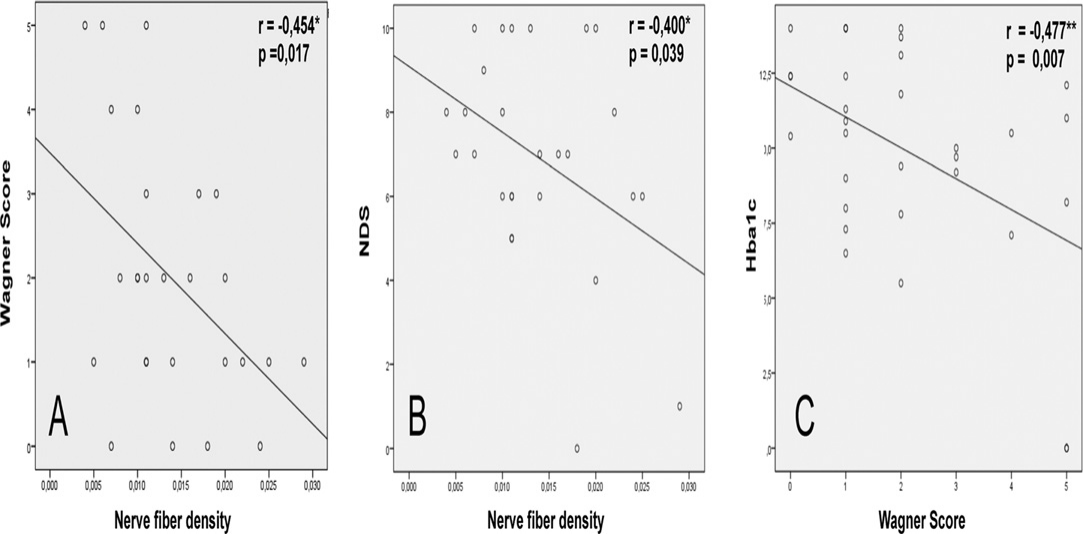
Correlation of study parameters. ** Correlation significant 0,01 (bilateral). * Correlation significant 0,05 (bilateral)

The analysis of the parameters (duration of DM, Hba1c, NFD, NDS, NSS, Wagner Score) revealed correlation between Wagner Score and NFD (p = 0.017, r = - 0,454), NDS and NFD (p = 0,039, r = - 0.400) as well as Wagner Score and HBa1c (p = 0,007, r = - 0.477) (Table 2, Fig. 2)

In quantitative terms in-vivo CLSM revealed only a decrease in average single fibre length in the DR subgroup compared with the NDR subgroup (p = 0.02). Comparison of the Wagner 1–5 group with DR and Wagner 0 group with NDR revealed a decrease in nerve fibre density (p = 0.04). Reductions in confocal parameters were noted both in Wagner 4–5 compared with Wagner 2–3 (nerve fibre density (p = 0.01); number of nerve branches (p = 0.003); connectivity points (p = 0.005)) and in Wagner 4–5 compared with Wagner 0–1 (nerve fibre density (p = 0.03); number of nerve branches (p = 0.01); connectivity points (p = 0.001)).

## Discussion

This study demonstrates for the first time the potential for diagnosing peripheral DN among DM patients in sub-Saharan Africa.

Diabetic foot ulceration is a global problem in DM, with a prevalence of about 11% in Africa [19] compared with 1% in Europe and North America. About 5% of all patients with type 2 DM have a history foot ulceration; the cumulative lifetime incidence is 15% [15, 16] and neuropathy is one of the most important features of foot ulceration. The prevalence of peripheral neuropathy varies from 10% to 36% [17], and the condition appears to be present in early diabetes due to the very low level of glycaemic control. The second contributory cause of foot ulceration is peripheral vascular disease (PVD), accounting for 35% of ulceration cases [18]. Interestingly, recent studies have shown that peripheral arterial disease in diabetic patients is associated with significantly higher amputation and mortality rates compared with non-diabetic patients [19]. The pathogenesis of foot ulceration is no doubt multifactorial (DN, PVD, walking barefoot, low hygiene standards, etc.) in the majority of cases. DM is believed to be a major risk factor for PVD.

In Europe the prevalence of PVD is about 9.5–13.6% among patients with type 2 DM compared with 4% in the general population [20]. Studies conducted in the UK have shown a 28% increase in PVD for every 1% increase in HbA_1c_ [21]. According to the literature, the prevalence of lower-limb arterial disease in Africa contributing to the development of diabetic foot syndrome ranges from 4% to 28% [17]. Moreover, poor hygiene standards coupled with low income exacerbate the wound care situation, resulting in the 12% lower-limb amputation rate in African patients with diabetic foot syndrome admitted to hospital [22].

The type 2 DM patients in our study were found to have a mean ABI of 1.1±0.3; in 16 out of 28 patients (57.1%) the ABI was ≥1.10 and in 5 out of 28 patients (17.8%) it was <0.9, pointing to medial arterial calcification rather than PVD as the cause of foot ulceration. 15/28 patients (53.6%) had moderate to severe diabetic foot ulceration. Very few studies have evaluated the possible correlation of ABI, peripheral diabetic neuropathy and diabetic retinopathy. While the association of high ABI levels with neuropathy has been confirmed [4, 23]. The validity of ABI measurements is affected by the presence of diabetes. Peripheral diabetic neuropathy decreases ABI sensitivity to 53% and specificity to 95% [20] due to secondary medial arterial calcification [23]. Nevertheless, high ABI levels are considered to be typical for diabetic patients and those with medial arterial calcification have a 1.7-fold increased risk of developing retinopathy [24].

In agreement with other authors [23], we noted a considerable discrepancy between extensive foot ulceration and only mild to moderate diabetic retinopathy in most of our patients. Our data show that 17 out of 28 patients (60.7%) have signs of DR with DM duration of 15.3 ± 7 years and HbA_1c_ of 11.2 ± 2.6%. This is consistent with a reported prevalence of 38% at 5 years and increasing to 55% in patients with disease duration >5 years [25]. Prevalence of 48% has been detected after 20 years of follow-up in Ethiopia [26] and where glucose control is poor (HbA_1c_ >8%) prevalence may even be as high as 77% [27]. The NDR group in our study showed significantly shorter diabetes duration without changes in HbA_1c_ level. An Australian study has reported a 2.5 odds ratio for the development of DR in the presence of PDN [28]. In line with this, our data show an increased NDS score in the DR group, emphasising the higher grade of PDN. Increased NDS as well as decreased corneal sensation were verified in the subgroup Wagner 1–5 and DR when compared with the subgroup Wagner 0 and NDR.

In our study we used in-vivo confocal microscopy to evaluate the small-fibre neuropathy. There were no differences in terms of morphology between the groups with and without DR, with the single exception of average single fibre length. A significant nerve fibre density decrease was detected in the subgroup group Wagner 1–5 and DR compared with the subgroup Wagner 0 and NDR.

At the same time there was found no difference in SBP morphology or corneal sensation in the subgroups with (DR) or without (NDR) diabetic retinopathy. This unexpected results are probably due to the small size of the group. On the other hand, Messmer et al. showed also the difference in corneal sensation between the healthy volunteers and diabetic patients. The authors also did not find any difference between the types of diabetic retinopathy or even between type 1 and 2 of diabetes in previous study [29].

Interestingly, comparable data were obtained in European patients with type 2 DM with PDN (HbA_1c_ 8.2 ± 1.6%; DM duration 15.1 ± 9.9 years; NDS 7.1 ± 2.6 points; NSS 7.1 ± 2.1 points): 39% of patients had DR, and although impairment of SBP morphology was also demonstrated in the NDR group, there were no significant differences between the NDR and DR groups [30]. In contrast, other groups have not reported differences in SBP morphology between healthy volunteers and DM patients without DR, but this was most probably due to the different image processing and analysis techniques used [29]. The modern development of confocal techniques and image evaluation implemented in the current study permits early detection of diabetic neuropathy in recently diagnosed (<2 years) type 2 DM patients [30].

The analysis of the parameters in the whole group revealed the correlation between Wagner Score and NFD (p = 0.017, r = - 0,454), NDS and NFD (p = 0,039, r = - 0.400) as well as Wagner Score and HBa1c (p = 0,007, r = - 0.477) (Fig. 2). Moreover, categorisation of the patients into three subgroups according to foot ulcer classification revealed a significant decrease in corneal sensation and NDS score from Wagner 0–1 to Wagner 2–3; a significant increase in ABI (indicative of medial arterial calcification); and a significant decrease in confocal parameters (nerve fibre density, nerve branches and connectivity points) from Wagner 0–1 to Wagner 4–5. A significant decrease in confocal parameters was also found from Wagner 2–3 to Wagner 4–5.

These suggest that the decrease in confocal parameters correlates with the grade of foot ulcer. The limitation of the current study is the absence of the age- and sex-matched control group. Nevertheless, we can extrapolate the result in Caucasian population to our study [29, 30, 32]. Numerous studies in DM patients have shown that the grade of PDN correlates well with SBP morphology. SBP changes (decreases in nerve density, number of branches, single nerve fibre length, tortuosity) have been found to correlate with established electrophysiological parameters as well as with the results of the skin biopsy [31,32].

Our data show the correlation of peripheral neuropathy with corneal neuropathy (Wagner Score and NDS to Nerve fiber density) as well as the Hba1c to Wagner score (Table 2, Fig. 2). Comparison of Wagner 1–5 with DR to Wagner 0 with NDR showed the differences in NDS, corneal sensation and Nerve fiber density. Analysis of Wagner 1–5 patients (that had some degree of ulcers) revealed only the difference in diabetes duration but not in corneal sensation or confocal parameters. The same is true to the comparison between the DR and NDR group in the whole study (Table 3). As supposed, peripheral neuropathy (NDS and Wagner score) correlates with corneal neuropathy (SBP morphology and corneal sensation) Our data are in line with other 15 studies dealing with various stages of peripheral neuropathy and morphology of SBP in confocal microscopy and corneal sensation analysed by Papanas in 2013. Thus, NFD has 56% sensitivity and 75% specificity for diabetic neuropathy; 63% sensitivity and 72% specificity for risk of foot ulceration [32].

In conclusion, the present study demonstrates the potential of in-vivo CLSM for evaluating peripheral diabetic neuropathy in sub-Saharan Africa. Our results confirm the primary diabetic aetiology of foot ulceration due to medial arterial calcification. The presence of PDN and PVD in conjunction with psychosocial factors is indicative of a multifactorial pathogenesis for foot ulceration. Patients with DR or with only superficial foot ulceration have a significantly increased NDS, while those with DR and advanced foot ulceration additionally show decreased corneal sensation and decreased nerve fibre density. The grade of diabetic foot syndrome correlates with corneal nerve changes and corneal sensation. With this in mind we hope to contribute to the development of programmes for the study, treatment and prevention of diabetes in Africa.
